# Ascorbic Acid Prevents Efavirenz-Induced Anxiety-Like Behavior and Brain Oxidative Stress in Zebrafish

**DOI:** 10.1155/omcl/8867221

**Published:** 2025-06-01

**Authors:** Emerson Feio Pinheiro, Norma Simone Santos da Costa, Milena Letícia Martins, Geovanna Ayami Saito, Nadyme Assad, Patrick Bruno Cardoso, Evander de Jesus Oliveira Batista, Suellen Alessandra Soares de Moraes, Adelaide da Conceição Fonseca Passos, Luana Ketlen Reis Leão, Amauri Gouveia, Karen Renata Herculano Matos Oliveira, Anderson Manoel Herculano

**Affiliations:** ^1^Laboratory of Experimental Neuropharmacology, Institute of Biological Sciences, Federal University of Pará, Belém, Brazil; ^2^Department of Psychobiology, Federal University of São Paulo, São Paulo, Brazil; ^3^Laboratory of Protozoology, Tropical Medicine Center, Federal University of Pará, Belém, Brazil; ^4^Laboratory of Neuroscience and Behavior, Federal University of Pará, Belém, Brazil

**Keywords:** anxiety-like behavior, ascorbic acid, efavirez, oxidative stress, zebrafish

## Abstract

Efavirenz (EFV) is a medication widely used for the treatment of HIV-positive patients. Several studies have demonstrated that the prolongate use of EFV can lead to the development of neurological diseases, such as panic syndrome, depression, and anxiety disorders. In this current study, we evaluate whether the ascorbic acid (AA) treatment can prevent anxiety-like behavior and brain oxidative stress induced by EFV treatment in zebrafish. Our data demonstrated that the EFV treatment induces anxiogenic-like behavior and intense lipid peroxidation in the zebrafish brain. The AA treatment was able to prevent both anxiogenic-like behavior and brain oxidative stress elicited by the EFV treatment. Therefore, our data provide robust evidence that the EFV induced anxiety-like behavior in zebrafish via a redox-dependent pathway and that AA treatment can minimize these adverse effects. Taken together, our preclinical study strongly suggests that the use of an AA-enriched diet can minimize the effects of EFV on the central nervous system (CNS) and improve the quality of life for patients undergoing EFV treatment.

## 1. Introduction

Efavirenz (EFV) is an antiretroviral medication widely used for the treatment of human immunodeficiency virus (HIV-1) infection [1, 2]. EFV inhibits the reverse transcriptase enzyme expressed in HIV-infected host cells, and its use confers significantly increased life expectancy of patients [3, 4]. Despite its high effectiveness as an antiviral medication, several studies have evidenced that the EFV treatment is commonly followed by adverse effects on the central nervous system (CNS) [3, 5, 6], including disturbances such as nightmares, mood changes, depression, and anxiety disorder [4]. Studies focusing on the prevention of EFV neurotoxic effects are highly relevant, given the impact on treatment adherence and patient quality of life.

EFV neurotoxicity on the brain has not been fully elucidated, however, previous reports have already demonstrated an association between EFV treatment and an increase of reactive oxygen species (ROS) in neuronal cells [7–9]. It is well documented that an elevation in the ROS levels in the brain can induce significant behavioral alterations, such as panic disorders, depression, and anxiety [10]. Other studies also show the crucial role of antioxidants in preventing the generation of anxiety behavior associated with brain oxidative stress [11]. Based on these findings, in this current study, we have hypothesized that the treatment with an antioxidant is able to prevent EFV-induced brain oxidative stress and anxiety-like behavior.

Ascorbic acid (AA) is an important natural antioxidant that can easily cross the blood–brain barrier, acting directly on the CNS [12, 13]. AA is present in several kinds of fruits, and it is used as a cofactor in the synthesis of hormones and neurotransmitters [14]. At a molecular level, AA is a potent ROS scavenger, preventing oxidative stress of biomolecules, such as lipid membranes [15]. Some reports have also shown that AA is able to prevent damage to the CNS by operating as a potent regulator of the redox status of the nervous tissues [16]. There are no studies describing whether the AA treatment can prevent the adverse effects of EFV on the brain, and characterization of this mechanism can significantly contribute to the development of safer therapies for HIV-positive patients.

Several studies have demonstrated that animal models represent a powerful tool for the testing of compounds able to exert an anxiolytic-like effect [17–20]. In this context, *Danio rerio* (zebrafish) stands out as a significant model for examining mechanisms underlying altered behaviors [21, 22]. Reports have indicated that molecules inducing anxiogenic and anxiolytic behaviors in humans have a similar effect in this animal model [23–26]. Furthermore, several toxicological studies have already used the zebrafish model to assess the impact of drugs in the redox homeostasis of different tissues, highlighting the relevance of this experimental model as an ideal translational research tool in the study of new therapeutic strategies [27]. Therefore, in this present study, we aimed to evaluate the neuroprotective effects of AA treatment against EFV-induced behavioral and biochemical changes in zebrafish.

## 2. Materials and Methods

### 2.1. Animals and Housing

Sixty-four *D. rerio* (zebrafish) short-fin from 3 to 4 months old, weighing 0.3 g (± 0.2) from both sexes (50:50 ratio), were purchased from a local supplier (Dular Aquários, Belém-Pará). Fish were housed with a controlled photoperiod of 14-h light/10-h dark, at a density of 1 animal per liter, for at least 2 weeks before the experiments began. All fish were fed once a day with commercial flake food (Tetra, Germany). All experimental procedures were made in compliance with the National Council of Animal Experimentation Control (CONCEA) and previously approved by the Ethics Committee in Research with Experimental Animals of the Federal University of Pará (CEPAE-UFPA: 213–14).

### 2.2. Drugs and Reagents

Drugs used in the current study were: antiretroviral drug EFV (C_14_H_9_NClF_3_O_2_) [28] and AA [16]. For the biochemical assay, N-methyl-2-phenylindole (NMFI), fetal bovine serum (FBS), methanesulfonic acid, and malondialdehyde (MDA) were all purchased from SIGMA–ALDRICH Corporation. For drug administration, the animals were individually cryoanesthetized in cold water at 4°C, followed by an intra-abdominal application (i.a) using a 10 µL Hamilton syringe as previously described by Assad et al. [18].

### 2.3. Groups and Treatments

**Figure 1:**
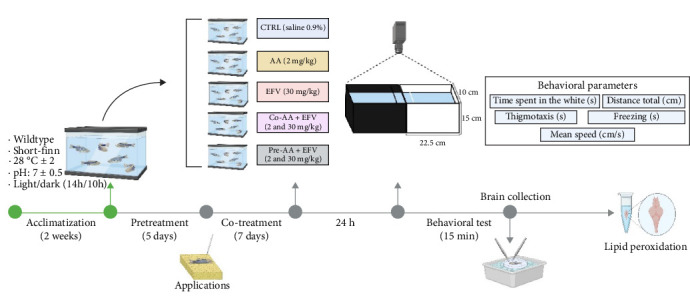
Timeline of the experimental procedures, starting with acclimatization, pharmacological applications followed by behavioral tests, and brain collection for the biochemical assays. (Illustration produced using BioRender.com).

The experimental groups were classified as: control (CTRL 0.9% saline solution), AA (2 mg/kg), EFV (30 mg/kg), co-treatment (AA-2 mg/kg + EFV-30 mg/kg), and pretreatment (AA-2 mg/kg + EFV-30 mg/kg), all drugs were injected via i.a (*n* = 10–14 per group). Treatment with AA + EFV was divided into co-treatment group (co-AA + EFV), in which AA was co-administered with EFV for 7 days, and the pretreatment group (pre-AA + EFV), in which AA was administered for 5 days before starting the 7 days of treatment with EFV, as described in Figure 1. During the applications, three animals were lost in the pre-AA + EFV group. In the biochemical assays, some samples were excluded from the analysis due to contamination, insufficient volume, or technical failures (EFV = 4; co-AA + EFV = 2).

### 2.4. Light/Dark Preference Test

In summary, 24 h after the last round of applications, zebrafish were individually transferred to a behavioral apparatus that consisted of an acrylic aquarium (15 × 45 × 10 cm) divided into two equal parts (light and dark), the animals were acclimated for 3 min and then freely explored both sides of the aquarium for 15 min, this protocol was adapted from Maximino [29]. All behavioral experiments were carried out between 8:00 am and 1:00 pm, with controlled lighting at an average of 500–600 Lx. The exploration of each animal was filmed and the videos were analyzed with the ZebTrack Software [30], the variables quantified were: time spent in the white (s): scototaxis behavior quantified by the individual's time spent in the light compartment; total distance (cm): distance traveled in centimeters that the animal covered during the behavioral test; thigmotaxis (s): the animal stays close to the periphery of the apparatus at a maximum distance of 2 cm from the walls; freezing (s): total time in seconds of freezing events, defined as complete cessation of movements with the exception of eye and opercula, with a minimum duration of 5 s; and mean speed (cm/s): mean speed of the animal during free exploration time.

### 2.5. Biochemical Assay

Brain oxidative stress in the CTRL and treated groups was measured by determining the MDA levels. This method consists of the quantification of molecular products caused by oxidative stress, allowing indirect quantification of the levels of lipid peroxidation in a tissue, using the colorimetric method described by Erdelmeier et al. [31]. After the cryoanesthesia, the animals were quickly decapitated with a sharp blade, their brain tissue was dissected and stored in a microtube containing 350 µL of tris-HCl buffer (pH 7.4) at −80°C until the time of analysis. This step was followed by tissue sonication and homogenization as previously described by Pinheiro et al. [32]. The homogenate was then centrifuged at 5600 rpm at 4°C for 10 min. MDA levels in the samples were determined by reaction at 45°C in the presence of 10 mM NMFI and methanesulfonic acid solution. Lipid peroxidation levels in the brains were analyzed based on standard curve concentrations of MDA, measured by absorbance at *λ* = 570 nm. MDA concentration in the samples was quantified in nmol per milligram of protein, and protein levels were determined by the Bradford method. The values were expressed as a percentage of the control.

### 2.6. Statistical Analysis

Data are presented as mean and standard of mean error (SEM) for behavioral analysis and percentage of controls for biochemical analysis. The normal distribution of data was determined by the Shapiro–Wilk test. The comparison between two groups was made through the Student's *t*-test, while differences between three or more groups were made by the analysis of variance (ANOVA) followed by Tukey or Bonferroni post hoc tests. All analyzes were performed using the GraphPad Prism software version 9.3.0 (GraphPad Software Inc., San Diego, CA, USA), with a significance level of *p*  < 0.05.

## 3. Results

### 3.1. EFV Treatment Induces Anxiety-Like Behavior

**Figure 2:**
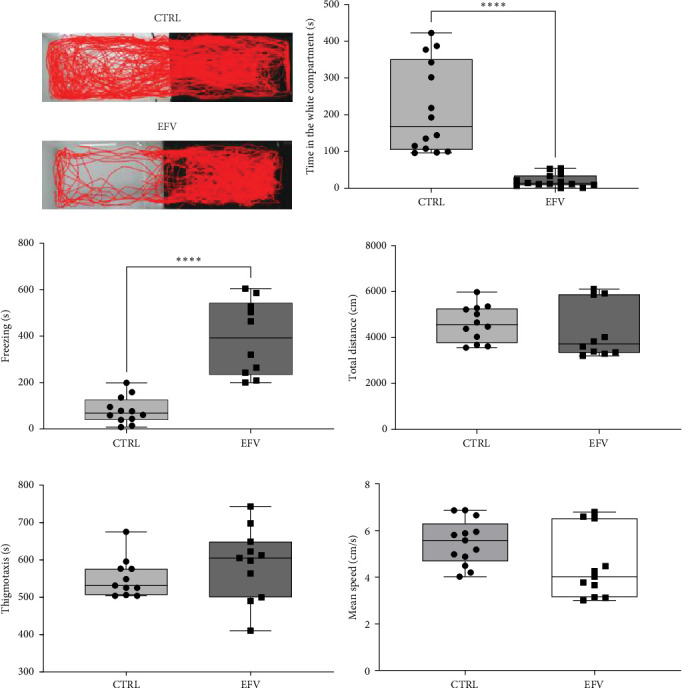
Effect of Efavirenz (EFV) treatment on zebrafish. (a) Locomotion in the dark and white side of aquarium, (b) time spent in the white compartment, (c) freezing, (d) total distance, (e) thigmotaxis, and (f) mean speed. Control saline solution (CTRL) 0.9% (*n* = 14) and EFV: 30 mg/kg (*n* = 14). Graphs represented as mean ± SEM and comparisons were made using Student's *t*-test. *⁣*^*∗∗∗∗*^*p*  < 0, 00001 vs. CTRL.

The results of the light/dark preference test demonstrated that the EFV treatment induced anxiety-like behavior in zebrafish. Thus, we observed that the EFV-treated group significantly reduced the time spent in the white compartment of the apparatus compared to the CTRL group (Figure 2b; *p*  < 0.0001). Additionally, animals treated with the EFV also showed an increase in freezing time compared to the CTRL group (Figure 2c; *p*  < 0.0001). However, when evaluating the total distance traveled (Figure 2d), thigmotaxis (Figure 2e), and mean speed (Figure 2f), no significant differences were observed between the EFV and CTRL groups.

### 3.2. The Oxidative Stress Induced by EFV in Zebrafish Brain is Prevented by the AA

**Figure 3:**
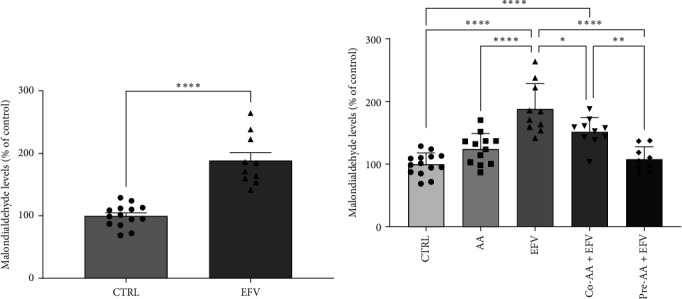
Lipid peroxidation in the zebrafish brain. Malondialdehyde levels in brain tissue of (a) control and EFV group and (b) co-treated and pretreated with AA + EFV. Control saline solution (CTRL) 0.9% (*n* = 14), AA: 2 mg/kg (*n* = 12), EFV: 30 mg/kg (*n* = 10), co-AA + EFV (*n* = 10), and pre-AA + EFV (*n* = 9). Data are expressed as a percentage of control ± SEM. For comparison between two groups, the Student's *t*-test was used and for more than two groups, one-way ANOVA was performed, followed by Tukey post hoc test. *⁣*^*∗∗∗∗*^*p*  < 0.00001, *⁣*^*∗∗∗*^*p*  < 0.0001, and *⁣*^*∗*^*p* ≤ 0.05.

To validate our hypothesis that the redox imbalance caused by the EFV treatment could induce anxiety-like behavior, we quantified the levels of MDA in the brains of these animals. Our data revealed that the EFV treatment caused an increase of approximately 88% in MDA levels compared to the CTRL group (Figure 3a; *p*  < 0.0001). In contrast, co-treatment and pretreatment with AA were effective in reducing MDA levels by approximately 35% (*p*=0.0249) and 77% (*p*  < 0.0001), respectively, compared to the EFV group (Figure 3b). However, we noted that the co-treated group did not return to redox homeostasis levels compared to the CTRL (*p*=0.0003) and pre-AA + EFV (*p*=0.0069) groups (Figure 3b). In the group treated only with AA, no significant effect on MDA levels was observed compared to the CTRL, co-treated, and pretreated groups.

### 3.3. AA Prevents EFV-Induced Anxiety-Like Behavior

**Figure 4:**
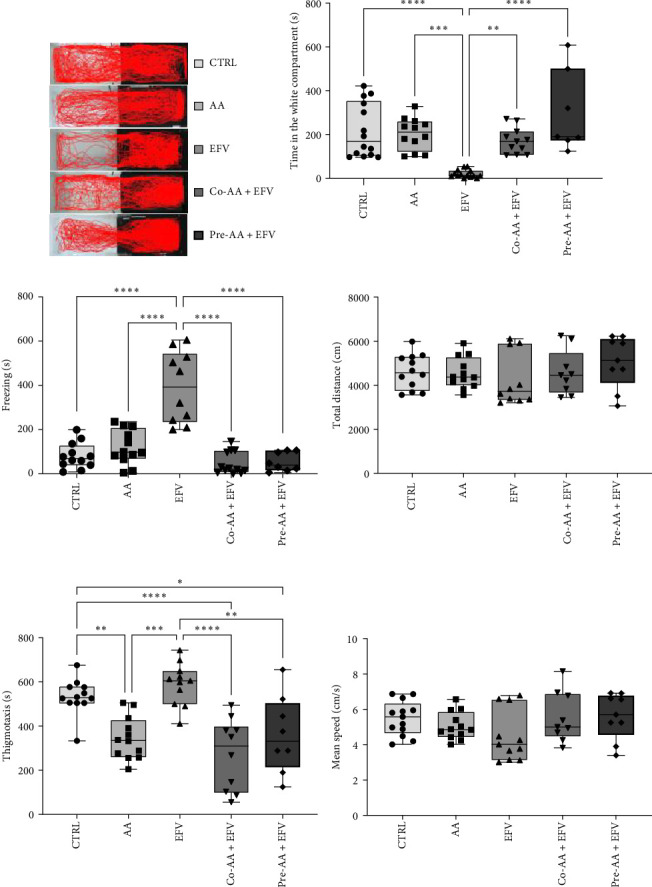
Effect of AA on zebrafish treated with EFV. (a) Locomotion in the dark and white side of aquarium, (b) time spent in the white compartment, (c) freezing, (d) total distance, (e) thigmotaxis, and (f) mean speed. Control saline solution (CTRL) 0.9% (*n* = 14), AA: 2 mg/kg (*n* = 12), EFV: 30 mg/kg (*n* = 14), co-AA + EFV (*n* = 12), and pre-AA + EFV (*n* = 9). The comparison between three or more groups was made by analysis of variance (ANOVA) followed by Bonferroni post hoc test. Graphs represented as mean ± SEM. *⁣*^*∗∗∗∗*^*p*  < 0, 00001, *⁣*^*∗∗∗*^*p*  < 0, 0001, and *⁣*^*∗∗*^*p*  < 0, 001.

Our data indicate that the treatment with AA prevented anxiety-like behavior induced by the EFV treatment. We observed that the groups receiving co-treatment (*p*=0.0085) and pretreatment (*p*  < 0.0001) with AA spent more time exploring the white compartment when compared to the group treated only with EFV (Figure 4a). Furthermore, the AA supplementation significantly reduced freezing time in the co-AA + EFV (*p*  < 0.0001) and pre-AA + EFV (*p*  < 0.0001) compared to the EFV group (Figure 4a). In the thigmotaxis parameter, the CTRL and EFV groups showed different behavior from the AA, co-AA + EFV, and pre-AA + EFV groups, demonstrating that these individuals did not avoid exploring the center of the apparatus (Figure 4e). However, we found no significant differences between groups regarding the total distance traveled (Figure 4d) and the mean speed (Figure 4f).

## 4. Discussion

In this study, we demonstrate that the EFV treatment induces neurochemical and behavioral changes in zebrafish. To translate anxiety into animal models, it is common to use aversive environments that mimic potential danger, such as the light/dark preference test [29]. For the first time, we show that EFV induces anxiety-like behaviors in adult zebrafish, as indicated by reduced exploration time in the light compartment and increased freezing time, both indicative of an anxiogenic state [33, 34]. These findings corroborate with previous studies that demonstrated that EFV (25 mg/kg and 50 mg/kg) reduced exploration time in the center of the open field and increased freezing time in mice subjected to sub-chronic treatment with this antiretroviral [28]. However, our results indicate that these behavioral changes are not associated with locomotor deficit, since the total distance traveled, and the mean speed were not changed. These results contrast with the findings of Zizioli et al. [35], who reported that EFV significantly affected morphological phenotype, reduced hatching rates, and increased apoptosis during the critical phase of neurodevelopment. The alterations impaired locomotor activity and mean swimming speed, suggesting that EFV exposure during the early hours of neurodevelopment is crucial for the expression of altered behaviors. This discrepancy can be explained by differences in the exposure period, as our experimental protocol involved adult zebrafish (3–4 months old), outside the critical window of neurodevelopment. This ensures that motor and neuromuscular circuits are already fully established, a process typically completed between 5 and 7 days postfertilization (dpf) [35]. These findings are consistent with clinical studies reporting neuropsychiatric events (anxiety and depressive behaviors) in patients who received high doses or underwent prolonged treatment with EFV [36].

Although mechanisms underlying the adverse effects of EFV have been extensively studied in in vitro and in vivo, they are not fully understood at a CNS level [3, 37]. Treatment with EFV can induce oxidative stress in various cell types, including liver cells, cardiovascular endothelial cells, glial, and neuronal cells [38, 39]. However, it is crucial for antiretrovirals to reach therapeutic levels in the CNS, as these organs are an important reservoir for viral replication [40]. In this context, there are few in vivo studies investigating the impact of EFV exposure on CNS redox homeostasis and its behavioral implications. In our study, we observed a significant increase of approximately 88% in brain MDA levels in the EFV-treated group. This neurotoxicity has been consistently reported in 40%–60% of patients treated with this antiretroviral, with these side effects being the main reason for therapy discontinuation/switching [41–43]. Edagha et al. [44], reported that this increase in MDA levels is associated with a decrease in the activity of antioxidant enzymes such, as glutathione peroxidase (GSH), aatalase (CAT), and superoxide dismutase (SOD), caused by exposure to different antiretrovirals, justifying the vulnerability of brain tissue to oxidative stress. The pharmacokinetics of EFV favor its neurotoxic action, as it is mainly metabolized in the liver through a process involving the cytochrome P450 system. Specifically, the isoform CYP450 2B6 (CYP2B6) catalyzes the hydroxylation of EFV into 8-hydroxyefavirenz, a metabolite closely linked to CNS side effects [3, 45].

Based on these results, we aimed to mitigate the redox imbalance in the CNS through the administration of the antioxidant AA. Thus, we observed a reduction of 33% and 77% in MDA levels in the co-treatment and pretreatment groups, respectively, compared to the EFV group. These data partially corroborate the findings of Edagha et al. [44], where the administration of antiretrovirals reduced the concentration of vitamin C in the brain tissue of rats. In another study, Suresh et al. [46] reported that patients with sustained infectious diseases, such as HIV-1, have significantly lower serum concentrations of antioxidants (e.g., AA) and elevated MDA concentration in the plasma. This phenomenon provides a plausible explanation for the success of AA supplementation in both experimental groups in our study, preserving redox homeostasis and avoiding elevated levels of lipid peroxidation in these animals' brains. It is important to highlight that the pretreatment group achieved a better performance in reducing MDA levels compared to the co-treatment group, demonstrating that vitamin C supplementation before initiating antiretroviral treatment may preserve redox homeostasis and strengthen the immune system, as suggested by Hughes et al. [47]. However, this biochemical difference did not immediately translate into changes in behavioral parameters between the co-AA + EFV and pre-AA + EFV groups. Our hypothesis is that the acute behavioral assessment, conducted 24 h after treatment completion, may not have captured the effects of the physiological alterations. Evidence suggests that biochemical adaptations may take time to translate into behavioral changes [48]. To better understand this relationship, further studies with long-term behavioral assessments after treatment are needed. This approach would help determine whether the reduction in lipid peroxidation in the pre-AA + EFV group leads to greater behavioral preservation over time compared to the co-AA + EFV group.

In the behavioral assessment, we observed that AA treatment was able to completely reverse anxiety-like behaviors. Individuals spent more time exploring the light compartment and showed no alterations in freezing behavior. Additionally, in thigmotaxis, the control and EFV groups exhibited longer thigmotaxis times compared to AA-treated groups. In the control group, this finding was expected, since the apparatus explores the animal's natural preference for staying close to the edges or sides of the apparatus (and avoiding the central open areas) [49]. In the EFV group, the increase in thigmotaxis was a consequence of the freezing time close to the edges of the apparatus, which occurs predominantly in the dark compartment [50]. In our analysis, the groups treated with AA presented similar thigmotaxis rates, which reinforces the hypothesis that AA exerts an anxiolytic effect when an isolated treatment, co-treatment, or pretreatment is implemented, as was shown by our results and supporting evidence from the literature [16]. This anxiolytic and neuroprotective effects of AA have been widely reported in the literature. Puty et al. [16], for example, demonstrated that AA prevented anxiety-like behavior and brain oxidative stress caused by methylmercury neurotoxicity in zebrafish. In another study with the same animal model, Paduraru et al. [51] showed that AA protected against the toxic effects of a lead and deltamethrin mixture. On rodents, AA supplementation prevented oxidative stress and promoted anxiolytic-like effects in rats exposed to cadmium chloride [52], and mice on a prolonged treatment with AA prevented anxiety-like behavior in the open field and light/dark preference tests [53]. In humans, a 14-day treatment with vitamin C resulted in lower blood pressure, faster cortisol recovery, and less anxiety in response to a psychologically stressful experience Brody et al. [54]. Previous studies have also shown that the neuropsychological adverse effects of EFV may be explained by direct interference with neurotransmitter systems, such as serotonergic, dopaminergic, GABAergic, and glutamatergic systems [28]. In the presence of free radicals, the brain's rich lipid composition favors lipid peroxidation, resulting in damage to membrane proteins, inactivating receptors, enzymes, and ion channels. As a result, oxidative stress can alter neurotransmission and brain function [55]. Together, these data demonstrate that AA is a viable alternative to maintain the brain redox homeostasis and prevent behavioral alterations evoked by the EFV treatment.

## 5. Conclusion

Therefore, our data provide robust evidence that the brain redox imbalance is an important link between EFV neurotoxicity and the behavioral changes observed in these animals. This suggests the possibility of using a diet rich in vitamin C, since this vitamin, even when supplemented in large doses, rarely presents toxicity, unlike fat-soluble vitamins. However, despite observing in this study that AA has the potential to regulate redox homeostasis and prevent anxiety-like behaviors, further research is needed to evaluate its effects on controlling disease progression in HIV-infected patients who need to use this antiretroviral.

## Data Availability

The data that support the findings of this study are available from the corresponding author upon reasonable request.
